# Invasive Micropapillary Carcinoma: A Rare Case of Male Breast Cancer

**DOI:** 10.7759/cureus.10571

**Published:** 2020-09-21

**Authors:** Emily A Coyle, Hiffsa Taj, Isin Comba, Jonathan Vasquez, Vania Zayat

**Affiliations:** 1 Pathology, University of Central Florida College of Medicine, Orlando, USA; 2 Internal Medicine, University of Central Florida College of Medicine, Orlando, USA; 3 Pathology, Orlando Veterans Affairs Medical Center, Orlando, USA

**Keywords:** male breast cancer, invasive carcinoma

## Abstract

Invasive micropapillary carcinoma (IMPC) is a rare form of breast cancer, accounting for 3.8-5.9% of all breast cancer types. Less than 1% of all breast cancer occurs in men and there have been only nine other cases of IMPC specifically in male patients reported in the literature. This case presents a 77-year-old Caucasian man who was found to have IMPC of the left breast after presenting for a painless mass in the left lower subareolar region. After a left modified radical mastectomy, the patient was found to have only one metastatic lymph node with his variant of IMPC being less aggressive requiring no further treatment other than annual surveillance with mammography.

## Introduction

Invasive micropapillary carcinoma (IMPC) of the breast is a rare variant of neoplasm which accounts for less than 2% of all invasive breast cancers [1]. In the case of male breast cancer, IMPC is even more unlikely with nine case reports detailing this subtype in male patients in the literature [2]. This subtype of carcinoma is characterized by neoplastic cells in a nested papillary pattern within clear spaces resembling lymphatic vessels [2,3]. Additionally, it demonstrates an aggressive clinical course with early lymphovascular invasion [2,4,5]. We present a case of a 77-year-old man with PT1c N1a Mx invasive micropapillary carcinoma of the left breast and review the current literature.

## Case presentation

A 77-year-old Caucasian man with a history of Hodgkin’s lymphoma treated with thymectomy and radiation at the age of 28 presented to the hospital with a chief complaint of a painless mass in his left breast of a few days’ duration. His other comorbidities included atrial fibrillation on anticoagulation, obstructive sleep apnea, and a remote history of pulmonary embolism. He denied any known family history of breast or other types of cancer.

**Figure 1:**
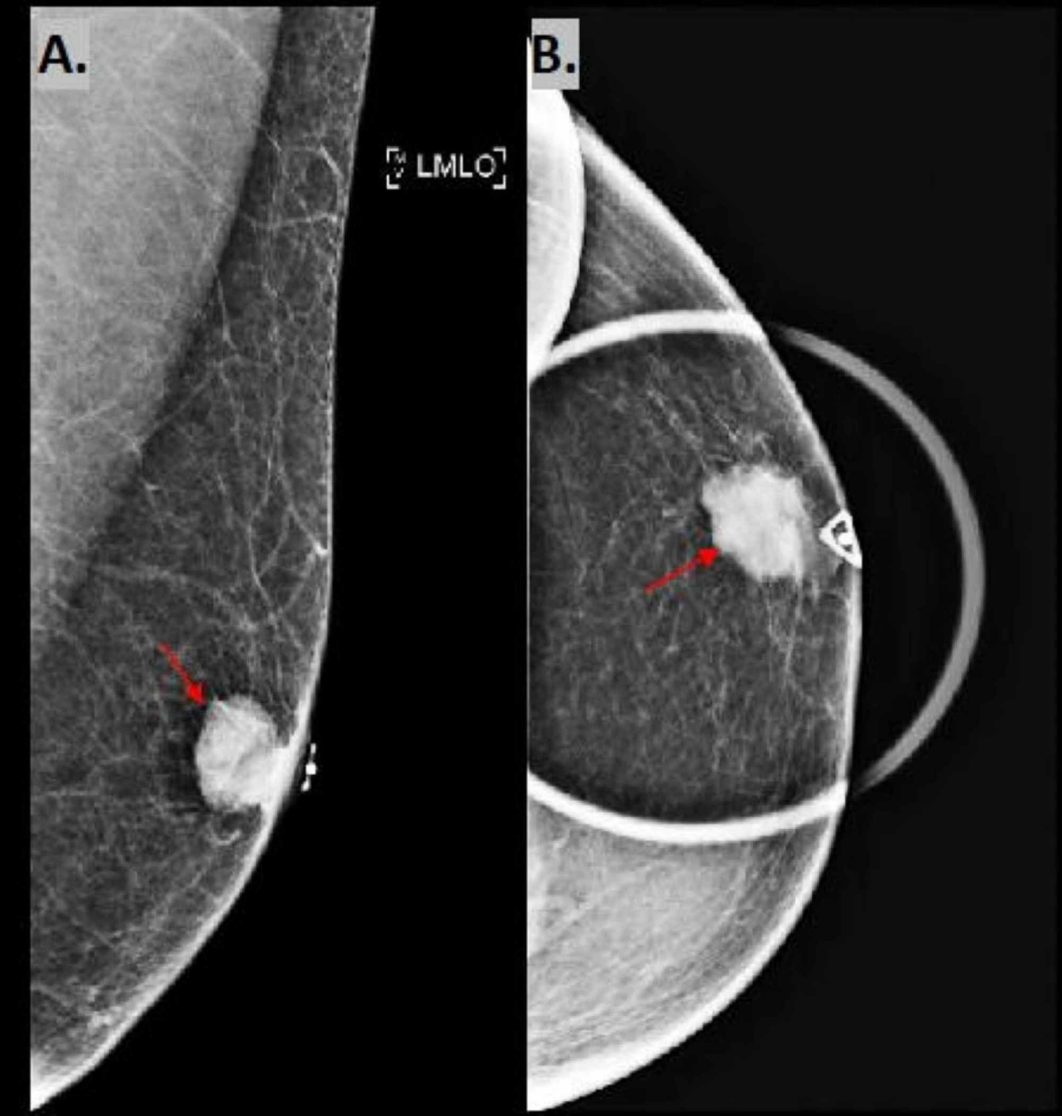
Mammography Mammography showing 1.8 cm x 1.8 cm x 1.8 cm left subareolar lobulated mass (red arrow).

**Figure 2:**
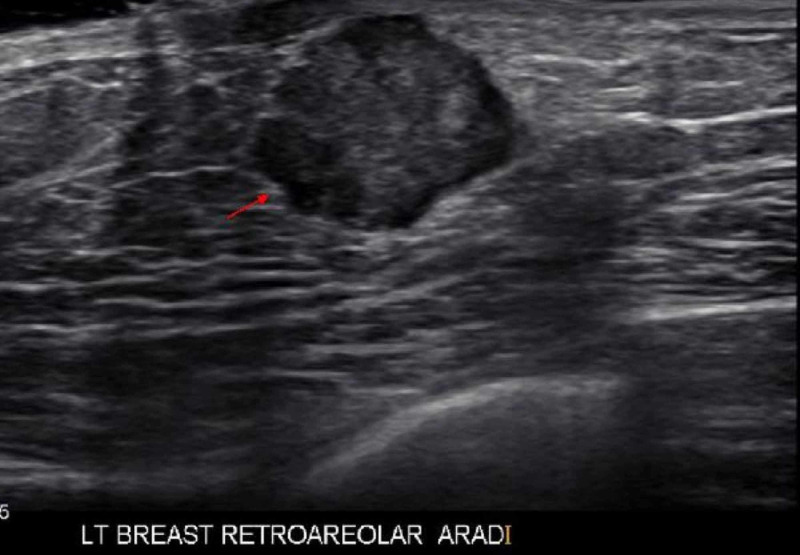
Ultrasound Left breast ultrasound showing hypoechoic vascular mass measuring 1.8 cm x 1.8 cm x 1.5 cm in size.

**Figure 3:**
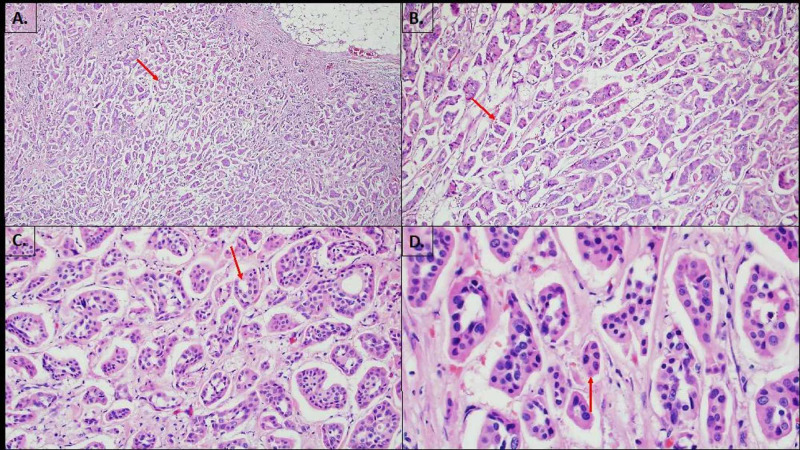
Needle core biopsy of left breast mass (A) Well-formed clusters of ductal epithelial cells sitting in the lacuna, (B) form papillary-like configuration. (C) Ductal epithelial cells create a central lumen without true fibrovascular cores. (D) Cells have abundant eosinophilic cytoplasm, oval to round nuclei and occasional prominent nucleoli.

On examination of the left breast, the area was non-erythematous with a palpable 1.5 cm firm, circular, painless mass in the left lower quadrant, subareolar region. The patient had a mammogram showing a 1.8 cm x 1.8 cm x 1.5 cm left subareolar lobulated mass highly suggestive of malignancy (BIRADS 5) (Figure 1). Left breast ultrasound showed a hypoechoic vascular mass with microlobular margins of similar size (Figure 2). Left axillary lymph nodes and right breast appeared normal at the time of imaging. A positron emission tomography (PET) scan for initial cancer screening was performed with no signs of nodal or distant metastasis. Needle core biopsy of the mass was performed and showed infiltrating ductal carcinoma (IDC) Grade 1 (mitotic rate: score 1, pleomorphism: score 2, tubules: score 3, total score: 5) (Figure 3). Immunohistochemical results were estrogen receptor (ER) positive (>90%), progesterone receptor (PR) positive (>90%), HER2/Neu 2+ equivocal with no amplification on fluorescence in situ hybrid (FISH), and E-Cadherin positive within tumor cells. The patient tested negative for mutations in the genes BRCA 1 and 2, TP53, PTEN, and PALB2.

The patient underwent a left modified radical mastectomy and a 1.7 x 1.5 cm solid, pale pink, well circumscribed mass was found subjacent to the nipple with a 0.4 cm central area of hemorrhage. Eight lymph nodes were dissected with one positive for metastatic carcinoma with three metastatic foci in the node. The largest focus measured 0.3 cm and was HER2 negative. Microscopic evaluation determined the patient to have invasive micropapillary carcinoma of the left breast and pathologic staging was evaluated at PT1c N1a Mx. Oncotype DX for breast recurrence was four, distance recurrence risk at nine years at 3%, and no apparent benefit of chemotherapy. After discussing the treatment options at length, the patient opted for surveillance with annual mammograms and no adjuvant chemotherapy, hormonal therapy, or radiation therapy.

## Discussion

Less than 1% of all breast cancer occurs in men and of that, invasive carcinoma of no special type (NST) is found in 84-90% and papillary carcinoma in 2-5% [1, 6]. Most frequently occurring in women aged 50-60 years old, IMPC accounts for 3.8-5.9% of all breast cancer cases. IMPC of the breast is an extremely rare entity in male patients with nine other cases reported in the literature. Burga et al. published a case series of 788 male breast cancer patients with a predominant histological type of pure infiltrating ductal carcinoma (84.7%) [7]. The few reports of IMPC in male patients demonstrate an average age of 69.8 years old [5]. This number is somewhat higher than that of women likely due to the overall rarity of breast cancer in male patients as well as a decreased awareness in that patient population [7].

IMPC was not distinguished as a separate type of invasive ductal carcinoma until 1993 when Siriaunkgul and Tavassoli recognized that both the morphological and molecular differences of these neoplastic cells gave them a stronger tendency to metastasize in pure form and when admixed with regular infiltrating duct carcinoma [2, 8]. Of those diagnosed with IMPC, almost 80% were found to have axillary lymph node metastases, commonly with three or more lymph nodes involved, demonstrating the aggressive and metastatic nature of this cancerous phenotype [3]. Contrary to the literature, our patient did not demonstrate significant distant metastasis with only one out of the eight axillary lymph nodes that were dissected positive for the disease. The patient’s immunohistochemical studies showed hormone positive and HER2/Neu negative cancer, indicating that it is a well differentiated cancer with good prognostic values. Like other types of breast cancer, triple negative (ER-, PR-, HER2/Neu-) disease is associated with higher grade, further advanced stage, and an increased propensity for lymphovascular invasion [9]. According to Gokce et al., hormone receptor negativity is hypothesized to exhibit more aggressive behavior in pure IMPC versus mixed cases, potentially accounting for our patient’s lack of significant metastasis compared to that expected from the literature [10]. Additionally, the initial stage at diagnosis has been implicated as another major factor for prognostic review [4]. The clinical stage of our patient was PT1c N1a Mx. This poses a better approach to multimodal treatment therapy that can improve recurrence rates and overall survival in the patient [4].

Our patient has a distant history of Hodgkin’s lymphoma (HL) as a young man for which he was treated with chest radiation. It has been found that breast cancer is the most common secondary malignancy for female survivors of HL, but there has been little to no discussion on the impact in males likely due to the relative rarity despite increasing incidence in the past 25 years. Factors such as young age at initial radiation exposure, radiation dose, radiation therapy field size, and time from treatment, all contribute to the likelihood of breast cancer emergence. This increased risk is usually observed starting 10 years post-therapy and beyond 25 years of follow-up, per Crump and Hodgson [11]. Information about the radiation therapy type and dosage for our patient was unavailable although it was noted that he was treated at 28 years old. Studies have shown that the risk of developing a secondary breast cancer in those evaluated post HL radiation therapy was highest with decreasing age at first treatment [11]. Unfortunately, these studies have not controlled for many variables that affect the female population versus the male population in question. These factors include the amount of breast tissue and lifetime exposure to hormones [7]. The patient’s history of radiation therapy could have been the initiating factor of his IMPC although not one specific carcinoma variant has been identified thus far as the typical secondary malignancy.

## Conclusions

Although breast cancer is described as the most common secondary malignancy to Hodgkin’s lymphoma, few cases have been reported in male patients. This report presents an elderly gentleman with IMPC of the breast almost 50 years after being treated with radiotherapy to the chest for HL. Unique to this patient was a less aggressive subtype of carcinoma allowing for treatment with radical mastectomy without adjuvant chemotherapy.
